# Hate the Sin, Love the Sinner: Examining the Role of Religiosity on Generation M’s Attitude Toward Purchasing Luxury Counterfeiting Products in Social Commerce

**DOI:** 10.3389/fpsyg.2022.927697

**Published:** 2022-09-29

**Authors:** Saqib Ali, Hasan Zahid, Nadeem Khalid, Petra Poulova, Minhas Akbar

**Affiliations:** ^1^Department of Management Sciences, COMSATS University Islamabad, Sahiwal, Pakistan; ^2^Faculty of Management Sciences, Riphah International University, Islamabad, Pakistan; ^3^Faculty of Business and Law, Anglia Ruskin University, Cambridge, United Kingdom; ^4^Department of Informatics and Quantitative Methods, Faculty of Informatics and Management, University of Hradec Králové, Hradec Králové, Czechia

**Keywords:** counterfeiting, religiosity, attitude, generation M, Pakistan

## Abstract

Counterfeiting has become a prevalent business worldwide, resulting in high losses for many businesses. Considerable attention has been paid to research an individual attitude toward purchasing luxury counterfeit products in the offline context. However, there is currently lesser-known literature on the given phenomenon in the context of social commerce. Moreover, researchers observed that counterfeiting consumption is associated with consumer ethical values or beliefs. Practitioners and researchers are keen to find those factors that affect consumers’ ethical consumption behavior to reduce pirated products’ demand. However, the role of religion in shaping ethical behavior is less documented in the counterfeiting context. Therefore, this study investigated the effect of religiosity on the counterfeiting of luxury products in Pakistan. A five-dimensional Islamic religiosity model was adopted to understand the consumption phenomena. For quantitative research, cross-sectional data were collected from the generation M of Pakistan through self-administrative questionnaires. A total of 394 valid responses from active online users were collected to empirically examine the conceptual model by employing the partial least square structural equation model (PLS-SEM). The results reveal that all five dimensions of religiosity negatively affect the attitude of generation M. Moreover, it is found that knowledge has the highest negative effect on attitude, followed by orthopraxis, experience, central duties, and basic duties. The study also explains the theoretical and practical implications of the research. Finally, limitations and future research were also discussed.

## Introduction

The virulence of the COVID-19 coronavirus pandemic has shaken the world (Omar et al., 2021). This pandemic hit hardly the world economies and societies. COVID-19 outbreak has volatility changed the working, communication, and buying behaviors (Di Crosta et al., 2021). The actions, attitudes, and purchasing patterns of the people differ in the pandemic situation from the normal times (Kamp et al., 2010). Accordingly, people have changed their buying behaviors in this COVID-19 pandemic situation around the globe especially in the developing countries (Alhaimer, 2022). In this tough economic situation of COVID-19, the buying patterns of consumers toward the necessities or non-necessities have been changed. Therefore, the COVID-19 situation has increased anxiety, fear, and depression and also has an impact on the spending and buying patterns (Di Crosta et al., 2021). According to Henderson and Schnall (2021), high intensity of fear, anxiety, and worry encouraged the harsher moral judgment toward the buying behaviors as compared to the lower intensity of the individuals. Counterfeiting has arisen as a major thread in all product categories (Khan-Mohammad, 2020). Counterfeits are manufactured illegally and infringe on legal rights (Singh et al., 2020). Counterfeit is considered a dishonest practice, inferior in quality, without seeking legal authorization, and economically harms the corporations around the globe (Yaakop et al., 2021). Counterfeiting is a growing problem and a hot topic for scholars, legislators, companies, and non-governmental organizations (NGOs) due to its enormous impact on economic development (Varela et al., 2021). According to the International Organization for Standardization ISO Report 2014, 5–7% of world trade accounted for counterfeit products, costing an estimation of 2.5 million jobs lost. In addition, 5% of the products imported by European countries were counterfeited (OECD/IEA, 2016). As per the report of Executive Board (2020), counterfeit products have a worth of $1.7 billion in 2015 and predicted $4.2 billion in the year 2022. Recent figures indicate that $4.5 trillion is traded in counterfeit products each year, with counterfeit luxury items accounting for 60–70% of the overall value (Fontana et al., 2019). According to the international black market figures, counterfeit shoes, clothing, and watch sales alone cost the industry $25 billion (Havocscope, 2022). The rising counterfeiting tendencies directly affect job losses, brand reputation, and national economies. For example, counterfeiting has resulted in the loss of nearly 2.5 million jobs globally (Davidson et al., 2019). Despite the enormous effort and resources committed to seizing counterfeit luxury products like handbags, watches, garments, jewelery, glasses, and shoes, the counterfeit industry continues to flourish in many under developing countries, including Pakistan, due to low income and lack of law and order situation (Niaz, 2020). Furthermore, due to ineffective law enforcement, consumers in developing countries, that is, Pakistan, are increasingly turning to counterfeit products (Niaz, 2020). Pakistan is among the top 10 nations globally from where the counterfeit products originate, and counterfeit goods are readily accessible and marketed openly throughout the country (Saeed and Sadiq Paracha, 2019). The presence and spread of counterfeiting are directly linked to customer demand, which is the primary driving factor behind every market. To reduce the demand for counterfeit luxury products, anti-counterfeiting measures must explore the underlying consumer motives for purchasing them.

Religion plays an extremely important role in guiding human behavior. Although, the association between religion and buying behavior was already established (Yaakop et al., 2021). Despite extensive research in this area, there are still several gaps in the literature that this study aims to contribute. First, there is currently a lack of research on counterfeiting that focuses on the role of religiosity in that phenomenon. Previous researchers have also confirmed the relationship between individuals’ religiosity and their attitudes toward various ethical and moral problems such as alcohol consumption (Russell et al., 2020), illicit drug use (Peltzer et al., 2016), viewing internet pornography (Perry and Whitehead, 2019), and tax evasion (Khalil and Sidani, 2020). However, few studies have looked at how religiosity influences counterfeiting behavior. Although there is no doubt that many researchers have studied the impact of religiosity on consumption behavior, the operationalization of the religiosity construct is still ambiguous (El-Menouar, 2014; Hassan, 2014; Ali et al., 2019a; Aslam et al., 2019). Moreover, a debate regarding religiosity as a unidimensional or multidimensional construct is discussed in several studies (El-Menouar, 2014; Islam and Chandrasekaran, 2015; Souiden and Rani, 2015; Felix and Braunsberger, 2016; Souiden et al., 2018), but the adoption of Glock (1972), multidimensional religiosity model, has been highly appreciated by most of the researchers (Niaz, 2020). Moreover, in the recent study of counterfeiting products, it is emphasized that researchers should use the multidimensional religiosity model in future studies for a better understanding of phenomena (Souiden et al., 2018). However, though researchers have found it useful and widely adopted, the Glock (1972) model has been criticized for its applicability in non-western or Islamic contexts (El-Menouar, 2014; Niaz, 2020). El-Menouar (2014) developed a multidimensional religiosity model in a non-western context by acknowledging this criticism. But, only a few studies have applied the El-Menouar (2014) multidimensional religiosity model in the Islamic and non-western contexts. So, the study’s first objective is to empirically test the El-Menouar (2014) religiosity model in a counterfeiting context.

Second, past research has found various significant predictors accountable for luxury counterfeit buying intention in the offline context but few in the online context (Islam et al., 2021). The internet makes purchasing counterfeits simpler for customers, as around 70% of counterfeit transactions occur online (Mooij, 2018). Practitioners have noticed the impact of counterfeit purchasing intention as a result of the internet in the digital revolution era in developing countries, that is, Pakistan (Niaz, 2020). In Pakistan, the sales of counterfeiting products have been rising for last few decades. Fewer effects have been taken to investigate the association of purchase intention of counterfeiting products (Cordell et al., 1996; Nurhayati and Hendar, 2020; Yaakop et al., 2021). Nurhayati and Bandung (2019) strongly recommended investigating the impact of religiosity and purchase intention. Moreover, religion is considered a strength to determine the purchase intention response of the consumers (Bupalan et al., 2019). Pakistan has the 4th most internet users per capita in Asia and the 10th most globally (Data Reportal, 2020). Pakistan has 76.38 million internet users; of which 44.10 million are online shoppers (Data Reportal, 2020). Moreover, Pakistan has 46.00 million social media users, which climbed by 9.0 million between 2020 and 2021, emphasizing the relevance of excessive smartphone usage and social commerce (S-commerce) (Data Reportal, 2021).

Third, the research mainly focused on generation M’s attitude toward counterfeiting products. Generation M refers to millennial Muslims of the new generation (Junaid et al., 2019). This Muslim generation has distinctive and unique needs, wants, and beliefs, making it a vital segment to study. Generation M is aware of Islamic preaching, portrayed in its consumption. They mainly focus on ethical consumption and avoid that behavior against Islam’s basic rules (Ali, 2017). Pakistan is one of the world’s youngest countries, having the largest population of generation M (Ali, 2017). Today Pakistan has the most significant percentage of young people recorded in history. According to research reports, with a 97% Muslim population, around 64% of the population’s age is less than 30, and 29% lie between 15 and 29 years (Kundi, 2018). Hence, it is essential to study generation M’s perspective regarding counterfeiting products in Pakistan, which is absent in the literature. So this study aims to fulfill this research gap.

## Literature Review

### Religiosity

Religion is an essential cultural value because it is unique, extensive, and dictates social institutes that profoundly affect the individual and society’s perception, belief, and behavior (Mokhlis, 2006). Theoretically, religious values can encourage and influence the behavior of individuals (Williams, 1979). Religious value differs from personal values because religious values are the moral concepts derived from scripture-based religious tradition, such as the Quran and Hadith for Muslims, deep-rooted in the individuals’ lives (Choi and Dooley, 2009). Religious activities play an essential role in shaping peoples’ approach to life, and religiosity is perceived as an essential factor that influences and motivates the beliefs, values, and practices of both individuals and society (Saroglou et al., 2004). Religiosity refers to “the religious belief a person practices in his life, and religiosity is the commitment to the religious practices” (Essoo and Dibb, 2004). Previous authors argue that religiosity is a continuous process (Beit-Hallahmi and Argyle, 1997) because religion may give meaning to the life of individuals, give a sense of achievements, security access to the social network and relationships, and also provides a set of standards for judging and guiding the actions of individuals (Rohrbaugh and Jessor, 1975). Previous researchers have confirmed the relationship between religiosity and attitudes of individuals toward different moral and ethical issues, such as the illegal use of the drug (Wagener et al., 2003; Mellor and Freeborn, 2011; Sanchez et al., 2011a), viewing obscene internet materials (Rostosky et al., 2004; Stack et al., 2004), students cheating (Barnett et al., 1996; Allmon et al., 2000), business ethics (Conroy and Emerson, 2004) Insider trading (Terpstra et al., 1993), alcohol consumption (Brown et al., 2001; Brechting et al., 2010a), and downloading (LaRose et al., 2005).

Nevertheless, there is still limited research addressing the role of religiousness in counterfeiting and digital piracy phenomena (Arli et al., 2017; Souiden et al., 2018). Previous studies measured religiosity through different scales and disagreed on religiosity as a unidimensional or multidimensional scale while measuring religiosity determinants (Souiden and Rani, 2015). But according to Glock and Huber, religiosity is measured through multidimensional, and the Glock religiosity model has an exploratory power for identifying different aspects of Muslim religiosity. These measuring instruments tend to have various problems, such as measuring religiosity through a unidimensional scale and interpreting research findings within Christian and western religiosity principles (Aji, 2018). Therefore, this study used (El-Menouar, 2014) model to measure religiosity because previous authors ignored the religiosity model which is developed by El-Menouar (2014), and this scale is more suitable and significant in the Muslim context, and the dimensions of this model are Basic Duties, Central Duties Experience, Knowledge, and Orthopraxy (Farrag and Hassan, 2015; Aji, 2018).

### The Role of Counterfeiting According to the Quran

According to the Quran verses, counterfeiting is prohibitive in Islam because Islam said any activity leading to such action is considered a fraud. Islam forbids all kinds of cheating and all deceiving acts, whether in buying and selling fraud or between people in any other matter. All Muslims are urged to be honest and trustworthy in everything they do in all situations.

يٰۤـاَيُّهَا الَّذِيۡنَ اٰمَنُوۡا لَا تَخُوۡنُوا اللّٰهَ وَالرَّسُوۡلَ وَتَخُوۡنُوۡۤا اَمٰنٰتِكُمۡ وَاَنۡـتُمۡ تَعۡلَمُوۡنَ‏

“O you, who have believed, do not deceive Allah and the Messenger or deceive your trusts while you know (the consequences)” (Quran 8:27).

وَلَا تُجَادِلْ عَنِ الَّذِيْنَ يَخْتَانُوْنَ اَنْفُسَهُمْ ۗ اِنَّ اللّٰهَ لَا يُحِبُّ مَنْ كَانَ خَوَّانًا اَثِيْمًاۙ

“And do not argue on behalf of those who deceive themselves. Indeed, Allah loves not one who is a habitually sinful deceiver” (Quran 4:107).

إِنَّ ٱ یَّأْمُرُكُمْ أَن تُؤَدُّوا۟ ٱلْأَمَنَتِ إِلَىٰٓ أَھْلِھَا

وَإِذَا حَكَمْتُم بَیْنَ ٱلنَّاسِ أَن تَحْكُمُوا۟ بِٱلْعَدْلِ إِۚنَّ ٱ نَِّعِمَّا یَعِظُكُم بِھِ إۦِٓۗنَّ ٱ كَّانَ سَمِیعًۢا بَصِیرًۭا

“Indeed, Allah orders you to render the trust to their owners, and when you judge between people to judge with justice. Excellent is that which Allah instructs you. Indeed, Allah is ever Hearing and seeing” (Quran 4:58)

إِنَّ ٱ یَُّدَفِعُ عَنِ ٱلَّذِینَ ءَامَنُوٓا۟ إِۗنَّ ٱ لَّا یُحِبُّ كُلَّ خَوَّانٍۢ كَفُورٍ

“Lo! Allah defended those who are true. Lo! Allah loved not each deceitful ingrate” (Quran 22:38).

وَأَوْفُوا۟ ٱلْكَیْلَ إِذَا كِلْتُمْ وَزِنُوا۟ بِٱلْقِسْطَاسِ ٱلْمُسْتَقِیمِ ذَۚلِكَ خَیْرٌۭ وَأَحْسَنُ تَأْوِیلًۭ

“And measure full when you measure. And weigh with an even balance. This is better, and its end is good” (Quran 17:35).

وَیْلٌ لِلْمُطَفِّفِینَ الَّذِینَ إِذَا اكْتَالُوا عَلَى النَّاسِ یَسْتَوْفُونَ وَإِذَا كَالُوھُمْ أَوْ وَزَنُوھُمْ یُخْسِر

“Woe to the defrauders who use short measures, who, when they measure [a commodity bought] from the people, take the full Measure, but diminish when they measure or weigh for them”(Quran 83:1,2,3).

یَٰٓأَیُّھَا ٱلَّذِینَ ءَامَنُوا۟ لَا تَأْكُلُوٓا۟ أَمْوَٰلَكُم بَیْنَكُم بِٱلْبَٰطِلِ إِلَّآ أَن تَكُونَ تِجَٰرَةً عَن

تَرَاضٍۢ مِّنكُمْ وَۚلَا تَقْتُلُوٓا۟ أَنفُسَكُمْ إِۚنَّ ٱ كَّانَ بِكُمْ رَحِیمًۭا

“O you who have believed, do not consume one another’s wealth unjustly but only (lawful) business by mutual consent. And do not kill yourselves (or one another). Indeed, Allah is to you ever merciful” (Quran 4:29).

وَلَا تَأْكُلُوٓا۟ أَمْوَلَكُم

بَیْنَكُم بِٱلْبَطِلِ وَتُدْلُوا۟ بِھَآ إِلَى ٱلْحُكَّامِ لِتَأْكُلُوا۟ فَرِیقًۭا مِّنْ أَمْوَلِ ٱلنَّاسِ بِٱلْإِثْمِ وَأَنتُمْ تَعْلَمُونَ

“And do not consume one another’s wealth unjustly or send it [in bribery] to the rulers in order that [they might aid] you [to] consume a portion of the wealth of the people in sin, while you know [it is unlawful]” (Quran 2:188).

### The Role of Counterfeiting, According to Hadith

Hadith is the saying of the Prophet Muhammad (PBUH). Hadith and Sunnah are both critical aspects of Islam. Hadith and Sunnah are two of Islam’s key aspects. Hadith plays an essential role in everyone’s life because it forms the person you are and who you have become along this life’s journey. Following the Prophet Muhammad’s hadith or Sunnah (PBUH), Allah’s ways and the messages he sent down from the heavens will be followed to help us achieve our goals.

Prophet Muhammad’s (PBUH) saying!

“The one who deceives is not one of us” (Sahih Muslim, Book 1, No 190)

“Don’t deceive someone who trusts you so you can’t cheat him” (Wasa’ il ul-Shia Vol. 12 pages 364).

He (PBUH) also said: “An honest and truthful Muslim trader shall be held with the martyrs on the Day of Resurrection.” (Ibn Majah, Book 12, Hadith 2222).

“If both parties were to speak the truth and explain the faults and attributes (of the goods), then they would be blessed in their transaction, and if they said lies and concealed anything, then their transaction’s blessings would be lost” (Bukhari and Muslim Resolution No. (101).

The Prophet has been recorded to state that it is not permitted to sell goods without making clear about everything, nor is it allowed for anybody who knows about the goods’ defects, not to mention them. An act of dishonesty is the same as cheating. One of the worst forms of fraud is dishonesty. A dishonest person is always likely to defraud others as often as possible such as misuse, false claim, intimate good, and so on.

### Basic Duties

The primary duties of Muslims contain all religious beliefs and attitudes. The Muslims believe that there is only one God (Allah), “Indeed, your God is one” (37:4), and Muhammad is the Prophet, “o Muhammad! They say ‘we testify that you are the last Messenger of Allah” (63:1). Religious teachings are based on three things, the “Quran” and the “Hadith” (Muhammad’s documented saying and acts), and the shari’a (Religious law) offers answers to all ethical questions (Rice, 2006). The Muslims believe that religion is not only faith but a systemic entity, a complete code of life. It offers guidance for its followers’ daily life, social, emotional, physical, and to some degree (Rice, 1999). Religion presumes two fundamental beliefs: faith in Allah and belief in the presence of another life. In other words, Muslims believe that their attitudes and acts in this life will affect their treatment in the hereafter (Tsalikis and Lassar, 2009). According to the Quran

“Rather, to Allah belong the hereafter and the first life” (Quran 53:25).

### Central Duties

The second dimension is the fulfillment of the core religious duties. These consist of following more or less the “five pillars of Islam” duties which consist of religious beliefs and practices (Aji, 2018): (1) “Shahadah–the profession of faith; that there is no other God than Allah and Muhammad is the messenger.” (2) Five prayers in a day “And you’re Lord says! Call upon me; I will respond to you. Indeed, those who disregard My worship will enter hell contemptible” (40:60). (3) A month of Ramadan fasting, “O you who have believed! Fasting is prescribed to you as it was prescribed to those before you, so you can learn to control yourself” (2:183). (4) Zakat is Islamic finance in which every individual has to contribute a particular portion of wealth each year to the community. “Who is that would loan Allah a goodly loan so He may multiply it for him many times over? And it is Allah who withholds and grants abundance, and to him, you will be returned” (2:245). (5) A journey of Makkah to perform Hajj or umrah. “Indeed, the first House of worship established for mankind was at Makkah-blesses and the guidance for the whole worlds” (3:96).

### Experience Dimension

The experience dimension includes people’s perceptions and practices of their faith (Rehman and Shahbaz Shabbir, 2010). Experience dimension is also known as mystical religiosity and it is defined as belief and practices in which Muslims seek the truth of God’s love and knowledge through God’s direct personal experience (Van Summeren, 2007), such as healing through prayer, “And when I am ill, it is God who cures me” (Quran26:80). And declare (O Muhammad) that (the Quran) is a guidance and healing for the believers (Quran41:44), protecting the Quran’s power, “Allah says in the Quran: never will we be struck except by what Allah has decreed for us; He is our protector. And upon Allah let the believers rely on” (Quran9:51), “And if an evil suggestion comes to you from Satan, then seek refuge in Allah. Indeed, he is hearing and knowing” (Quran7:200), brotherhood, “the believers are but brothers, so make reconciliation between and fear Allah that you may receive mercy” (Quran49:10), and reward behavior, “Is the reward of goodness anything but goodness” (Quran55:60). Indeed, Allah does not do injustice, even as much as an atoms weight; while there is a good deed, He multiplies it and gives from Himself a great reward (Quran4:40) and punish behavior, “As to those who reject faith, I will punish them in severe a punishment in this world and in the hereafter, nor will they have anyone to help”(Quran3:56) (Van Summeren, 2007; Aji, 2018).

### Knowledge Dimension

The knowledge aspect involves knowledge of the person about religion. The contents of the Quran and Sunnah are usually the primary source of Islamic knowledge. Believers are expected to know a minimum of these contents. Muslims call for the in-depth understanding of equality for all human beings, a strong sense of brotherhood, good or bad deeds, including morality, modesty, humility, trustworthiness, duty, justice, patience, fairness, and tolerance, ethics in dealing, care, empathy, and compassion (Rice, 1999; Ali and Weir, 2005).

“He also says, we have revealed to you the book which clarifies every matter” (Quran 16:89).

“Indeed this Quran guides to the path which is clearer and straighter than any other” (Quran, 17:9).

### Orthopraxis Dimension

The Orthopraxis dimension of religiosity plays an important and distinct role in Muslim religiosity (Aji, 2018). Orthopraxis define as Muslims maintaining and practicing religious beliefs, practices, norms, and values in their daily life (Van Summeren, 2007), such as halal/haram, “O you who believe! Eat of the good things wherewith we have provided you, and render thanks to Allah if it is (indeed) He whom you worship” (Quran 2:127), the ban on interest “But Allah has permitted trade and has forbidden interest” (Quran 2:275), gender relationship, “And come not near unto adultery. Lo! It is an immorality and evil way” (Quran17:32), music (El-Menouar and Stiftung, 2014), “For the harshest of sounds, without doubt, is the braying of the ass” (Quran31:19), and differs from believer to believer, and the Muslim community fully accepts these examples as religious norms (Khadduri, 1989). The author found that people who are more orthodox strictly follow the religious norms and values (Aji, 2018).

H1(a): Basic Duties dimension of religiosity negatively affect consumers’ attitude toward purchasing luxury counterfeit products.

H1(b): Central Duties dimension of religiosity negatively affects consumers’ attitude toward purchasing luxury counterfeit products.

H1(c): The experience dimension of religiosity negatively affects consumers’ attitudes toward purchasing luxury counterfeit products.

H1(d): The knowledge dimension of religiosity negatively affects consumers’ attitudes toward purchasing luxury counterfeit products.

H1(e): Orthopraxis dimension of religiosity negatively affect consumers’ attitude toward purchasing luxury counterfeit products.

## Materials and Methods

### Survey Design

A survey method was employed to validate the conceptual model because the quantitative research method predicts individual responses and examines the relationship between constructs (Newsted et al., 1998). Many researchers in the past have employed survey methods to study behavior in the field of social commerce (Lin et al., 2018; Liu et al., 2019; Sheikh et al., 2019). An online survey was used to collect data for this study. To manage the various responses from the respondents, the mail chimp software is used to administer all the online responses. The prospective respondents were Pakistani social media users who had at least one account on any social media platform (Facebook, Twitter, Instagram, or others). An online survey is deemed advantageous since it is less costly and gives timely replies with a broad geographic reach (Denscombe, 2006). In addition, by using an online survey, consistency between the study and data gathering contexts may be maintained (Liu et al., 2016). As a result, we believe that a survey method is the most appropriate approach for this study.

### Measurement Scale

A 7-point Likert scale was used to measure all these constructs that ranged from strongly disagree = 1 to strongly agree = 7. A quantitative approach using the survey questionnaire method was used to examine the influence of religiosity on Pakistani Muslim youth’s attitude toward counterfeit products. The questionnaire comprises two parts. Section A will be the attitude (5 items) (Ting et al., 2016), basic duties (3 items), central duties (6 items), knowledge (3 items), experience (4 items), and orthodox (6 items) (Aji, 2018) and section B includes demographics of age, gender, education, and income level.

### Data Collection

In the current study, the non-probability sampling technique of purposive sampling was employed. It is suitable and more appropriate when the sampling frame is not available and population is unknown (Ali et al., 2019b). Moreover, non-probability sampling techniques are suitable for theoretical generalization. Therefore, the purposive sampling technique was adopted. The data were collected from four universities in two major cities of Pakistan, namely Lahore and Multan, during the Spring 2021 semester. In coordination with the university’s student affairs office, a survey questionnaire was sent to students through their email accounts. The final questionnaire and a covering letter were sent out by email to the students who requested to participate in this research. We sent 600 questionnaires through email and received 427 responses, 394 of which were usable. As a result, the response rate was 65.67%. All of the students who were asked to fill out the questionnaire had an account with at least one of Pakistan’s social media websites. Tables 1, 2 shows the respondents’ demographics.

**TABLE 1:** Characteristics of respondents.

	Characteristics	Frequency	Percentage
Gender	Male	227	57.61
	Female	167	42.39
Age	18–21	104	26.40
	22–25	148	37.56
	26–29	89	22.59
	30–over	53	13.45
Education	Undergraduate Graduates Postgraduate	176 153 65	44.67 38.83 16.50

**TABLE 2:** Measurement model.

Constructs	Items	Loadings	AVE	CR
**Basic religiosity**				
	BASR1	0.693	0.510	0.862
	BASR2	0.759		
	BASR3	0.712		
	BASR4	0.716		
	BASR5	0.667		
	BASR6	0.736		
**Central duties**				
	CEND1	0.855	0.712	0.881
	CEND2	0.863		
	CEND3	0.813		
**Experience**				
	EXPE1	0.934	0.640	0.839
	EXPE2	0.789		
	EXPE3	0.652		
**Knowledge**				
	KNOW1	0.861	0.656	0.851
	KNOW2	0.747		
	KNOW3	0.818		
**Orthopraxis**				
	ORTH1	0.828	0.614	0.823
	ORTH2	0.887		
	ORTH3	0.607		
**Attitude**				
	ATTI1	0.707	0.565	0.838
	ATTI2	0.713		
	ATTI3	0.788		
	ATTI4	0.794		
**Basic religiosity**				
	BASR1	0.693	0.510	0.862
	BASR2	0.759		
	BASR3	0.712		
	BASR4	0.716		
	BASR5	0.667		
	BASR6	0.736		
**Central duties**				
	CEND1	0.855	0.712	0.881
	CEND2	0.863		
	CEND3	0.813		
**Experience**				
	EXPE1	0.934	0.640	0.839
	EXPE2	0.789		
	EXPE3	0.652		
**Knowledge**				
	KNOW1	0.861	0.656	0.851
	KNOW2	0.747		
	KNOW3	0.818		
**Orthopraxis**				
	ORTH1	0.828	0.614	0.823
	ORTH2	0.887		
	ORTH3	0.607		
**Attitude**				
	ATTI1	0.707	0.565	0.838
	ATTI2	0.713		
	ATTI3	0.788		
	ATTI4	0.794		

## Results

Structural Equation Modeling (SEM) has an advantage for statistical analysis in terms of efficiency, accuracy, and convenience over traditional multivariate statistical techniques (Usakli and Kucukergin, 2018). Moreover, SEM helps to examine both explanatory and confirmatory analysis. Structural equation modeling (SEM) has two well-known methods: covariance-based SEM (CB-SEM) and variance-based SEM (VB-SEM) (Hoyle, 1999). Partial least square (PLS-SEM) is used in this study to analyze the theoretical framework because the partial least-square SEM approach presents findings in two parts: the measurement model and the structural model. The measurement model (validity and reliability) and the structural model (testing the hypothesized relation between variables) were tested.

### Measurement Model

**FIGURE 1:**
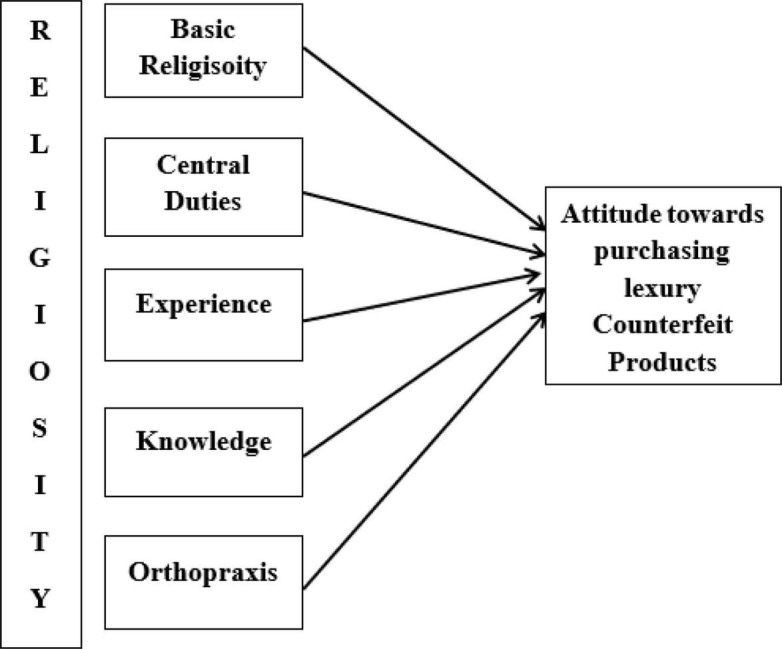
Theoretical framework.

The measurement model is used to observe the relationship between observed data and latent variables, and it also explains the calculation of variables. The advantage of this model was to assess the valuation of validity and reliability test. Construct reliability is measured using outer loading, and internal consistency of reliability was measured through composite reliability. Moreover, convergent reliability is measured through the average variance extracted (AVE) (Hair et al., 2014). To ascertain the items’ reliability, the out loadings should be above the suggested value of 0.50 (Hair et al., 2014), and the composite reliability should exceed the recommended value of 0.7. The average variance extracted (AVE) value is above the threshold value of 0.50 (Anderson and Gerbing, 1988). As shown in Table 2 (Figure 1), all outer loadings were found to be above the threshold values, the composite reliability (CR) value was higher than 0.70, and average extracted variance (AVE) scores were greater than 0.50.

Next discriminating validity, Heterotrait-Monotrait correlation ratio (HTMT) TEST, was used to measure discriminating validity because it is more potent than other methods. According to Kline (2015), the HTMT value should be less than 0.85, but Gold argues that the HTMT value should be less than 0.90 to affirm discriminant validity (Gold et al., 2001). All HTMT values are below the threshold value (see Table 3).

**TABLE 3:** Discriminant validity [Heterotrait-Monotrait (HTMT) criterion].

	Attitude	Basic religiosity	Central duties	Experience	Knowledge	Orthopraxis
**Attitude**						
Basic religiosity	0.571					
Central duties	0.640	0.591				
Experience	0.223	0.121	0.178			
Knowledge	0.889	0.585	0.694	0.095		
Orthopraxis	0.673	0.407	0.626	0.161	0.593	
						

### Structure Model

**FIGURE 2:**
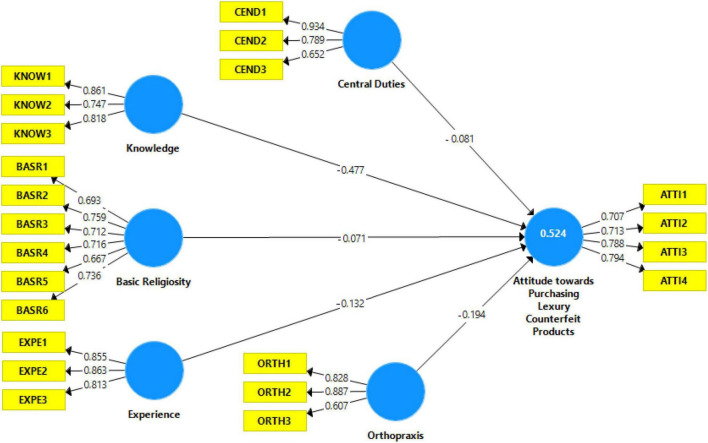
Structural model (bootstrapping results).

Once the measuring model has been confirmed as reliable and valid, the second step is to examine the structural model. The structural model involves the significance of path coefficient, *t*-values, coefficient of determination (R2), effect size (f2), and predictive relevance (Q2). Using the bootstrapping method (5,000 resample), path coefficient significance was measured. The results indicate that all hypotheses are accepted and significant toward individuals’ attitudes to purchasing counterfeit products. However, findings suggested that the knowledge dimension has the most significant negative impact on attitude to use luxury counter fitting products (47%), followed by orthopraxis dimension (19%), experience dimension (13%), central duties (8%), and basic religiosity (7%) (see Table 4). The R2 of attitude is 0.524, which indicates that 52% variation in attitude to use luxury counterfeiting products is due to these five religiosity dimensions, which is a moderate effect as per indication by Hair et al. (2011) who suggested that R2 values of 0.25, 0.50, and 0.75 are considered as a week, moderate, and substantial. As per Cohen (1998), effect size ranges from small to large because 0.02 is deemed to be small, 0.15 as a medium, and 0.35 as a strong effect. Moreover, this study does not rely only on R2 but also checks the predictive relevance in the recommended range, that is, 0.204 > 0 (Figure 2).

**TABLE 4:** Hypotheses testing.

Hypothesis	Relationship	Path-coefficient	Std. error	*t*-value	*p*-value	Supported	f2	R2	Q2	SRMR
H1	BASRATTI	–0.071	0.034	1.982	0.018	Yes	0.008	0.524	0.204	0.076
H2	CENDATTI	–0.081	0.041	2.1051	0.024	Yes	0.010			
H3	EXPEATTI	–0.132	0.035	3.810	0.000	Yes	0.026			
H4	KNOWATTI	–0.477	0.040	11.885	0.000	Yes	0.302			
H5	ORTHATTI	–0.194	0.039	4.981	0.000	Yes	0.057			

## Discussion

Counterfeiting is a centuries-old crime, the proliferation of counterfeit products has become a global phenomenon, and indeed is a serious business issue all over the nation (Wilcox et al., 2009; Chaudhry and Stumpf, 2011). It’s fascinating how religion, culture, and counterfeit products interact. However, there is a lack of academic research investigating the influence of religiosity and attitude toward counterfeit products, especially among Muslim consumers (Arli et al., 2017; Souiden et al., 2018). Because of the lack of academic research and gap in the literature, this study’s primary purpose is to examine how Muslim religious commitment influences the attitude toward counterfeit products. Data analysis confirmed that five religiosity dimensions such as basic duties, central duties, experience, knowledge, and orthopraxis directly influence the individuals’ attitudes toward counterfeit products.

The knowledge dimension means the person’s knowledge of the content of Islam (Waardenburg, 2002). The results show that the Knowledge dimension has the highest negative impact on consumers’ attitudes to buy luxury counterfeiting products in Pakistan (Beta = -0.477, *p* > 0.05). The highest impact of the knowledge dimension is in line with the expectations that more knowledge and study of basic tenets and scriptures of Islam influence the followers to act accordingly. This finding also collaborates with the previous studies (Farrag and Hassan, 2015; Briliana and Mursito, 2017), who suggested that the contents of the Quran and Hadith completely guide the followers in every aspect of life and it also instructs them on what is right and wrong (Rohrbaugh and Jessor, 1975), and when the individual has more knowledge about the content of Quran and Hadith than the individual behaves in a relatively more.

Orthopraxis was the second-highest effective dimension of attitude toward counterfeiting product usage in Pakistan. Orthopraxis means the Muslims uphold the religious belief and follow norms and values in their daily lives (Van Summeren, 2007). The results indicated that orthopraxis significantly negatively impacts consumers’ attitudes toward counterfeiting products in Pakistan (Beta = -0.194, *p* > 0.05). The results are also in agreement with previous research (Farrag and Hassan, 2015; Souiden et al., 2018), and the possible reason for these results could be the orthodox person who strictly follows the religion and did not compromise on religious norms (gender segregation, avoid shaking of hands, and avoiding listening to music) (Aji, 2018). The orthodox Muslim has more developed in fear of divine punishment. They are more afraid of being in haram than less likely to be involved in unethical and illegal activities. It has a significant negative effect on attitudes toward counterfeit products.

The experience dimension is also called mystical religion, and this may include feelings, emotions, and knowledge generated from some experience with the ultimate divine truth (Van Summeren, 2007). Empirical findings suggested that the experience dimension has a moderate negative impact on available dimensions on the consumer’s attitude toward counterfeiting luxury products in Pakistan (Beta = -0.132, *p* > 0.05). One plausible explanation for the significant results is that when the individual is more afraid of the creator, they abide by what is considered good (halal) and avoid what is deemed bad (haram), which can be seen in their practices. Muslims follow the ALLAH instructions because they know that their excellent behavior rewards them and their bad behavior punished them in the hereafter (Souiden and Rani, 2015). These experiences of instructions motivate consumers to avoid those products that are not legal, for instance, counterfeiting luxury products.

Central duties include religious practices like prayers, dedication, and all the things a person is doing to fulfill their religious commitment. The person follows all the spiritual approaches at the collective level (Edwards and Carpenter, 2013). H2 claims that central duties directly influence the attitude toward purchasing counterfeit products. Results of central duties (Beta = −0.081, *p* > 0.05) show a negative association of religious duties with attitude toward counterfeit products with a weak effect. The results are consistent with the earlier study. Previous studies also suggest that a person with strong religious commitment is less likely to engage in unethical behavior such as illegal use of the drug (Wagener et al., 2003; Mellor and Freeborn, 2011; Sanchez et al., 2011b), viewing the internet obscene materials (Wagener et al., 2003; Stack et al., 2004), students cheating (Barnett et al., 1996; Allmon et al., 2000), business ethics (Conroy and Emerson, 2004), insider trading (Terpstra et al., 1993), and alcohol consumption (Brown et al., 2001; Brechting et al., 2010b). The more religious people respect the religion, follow, and practice it (Souiden and Rani, 2015), and their submission must be evident in the individuals’ actions.

Basic duties simply mean the core belief of every individual, individuals committed to these beliefs on the individual level (Aji, 2018), and basic duties directly influence the attitude toward the purchase of counterfeit products. The results of this study confirm that basic duties (Beta = −0.071, *p* > 0.05) have a weak negative relationship with attitude toward the purchase of counterfeit products. Some previous researches also validate the finding of the study. Basic duties are interconnected with the core or fundamental values of Islam, which differentiate Muslims from non-Muslims. The main reasons for the results are the previous author characterized these beliefs into three types. The first type of belief explains the exitance of the divine, the second type of belief explains the purpose of the divine, and the third describes the ethical structure of the whole region (Hassan, 2007). However, basic religiosity does not guarantee the following of rituals and experiences, and it is the individual’s level of religiosity that varies. That’s why it is the weakest dimension affecting consumer attitude in Pakistan.

## Practical AD Theoretical Implications of the Study

The study has investigated the effect of religiosity on luxury counterfeited products in Pakistan. The study has contributed theoretically in many ways. Researchers have confirmed the relationship between attitude and religiosity of individuals toward different moral and ethical issues, such as the drug’s illegal use. Nevertheless, there is little literature on the relationship between religiosity and counterfeit/pirated products. So, studying the effect of religiosity will enhance the understanding of the phenomena. Second, the study has adopted a five-dimensional Muslim religiosity model to understand Pakistan consumers’ behavior. However, previous researchers have adapted the translated Glock (1972) model to the Muslim context, with many limitations explaining Muslim consumers’ religiosity. So, the adoption of the Islamic religiosity model in the counterfeiting context has added new insights to the literature. Third, the study has targeted generation M for the research. Generation M is, in particular, Muslim consumers who have unique desires, consumption, and living patterns. Studying generation M in Pakistan has contributed as Pakistan having one of the largest young populations. Moreover, these consumers care about the ethical and moral decision-making. However, the literature regarding generation M consumption behavior of counterfeiting products was not found. So, this research has fulfilled this gap in the literature that was absent. Finally, our research contributes to a better understanding of the online consumers’ purchasing behavior in S-commerce by focusing on a crucial purchasing experience: counterfeit purchase intention. There was a lot of research on why consumers buy counterfeit products in the offline context in the past. However, previous research has not looked at the determinants influencing consumers to purchase counterfeiting products in s-commerce.

### Managerial Implications

This study’s results help the managers, particularly those who deal in the market of Islamic countries. First, this study mentioned that religiosity is the main factor that predicts attitude toward counterfeit products. The main problem is that people do not see themselves as unethical in buying counterfeit products. In this regard, the Muslim country used religious appeal. However, various unethical practices, including corruption and digital piracy, exist in Pakistan, so spiritual appeal plays a vital role in changing young consumers’ perception because 87% of Pakistani people like to see themselves first as Muslim. So, marketers must use the Quran and Hadith in their religious appeal, which will allow them to buy the original products instead of counterfeiting. While making advertisements and marketing campaigns, the marketers must use these sentences, such as Allah said! Do not deceive others; Allah wants justice, which helps them to provoke and motivate them to buy legal or original products. Second, the managers and marketers can change these attitudes by educating and providing awareness about the side effects of using counterfeit products and original brands by providing information through blogs, emails, websites, social media, and so on, about the logo and trademarks, and their packaging. Original manufacturers also make time to time changes in the product’s designs and packaging to attract and retain consumers. Hence, customers are more conscious while purchasing products.

### Organizational and Institutional Implications

The governments, politicians, and news organizations should cooperate and reinforce “anti-counterfeiting organizations,” such as “Pakistan’s anti-counterfeiting agency, Pakistan’s Standard” and “Quality Control Authority,” in Pakistan to prevent counterfeiting by sharing the experience of damage and harmful effects of counterfeit products and counterfeit business. Also, enact strict rules and regulations and levy sanctions or prosecute producers, distributors, and customers, impose penalties and punishments on buyers and sellers if they have been found doing counterfeit-related activities, and finally to prevent counterfeit purchases in the online context; legislators and IT experts should rigorously monitor online retailers’ activities and establish an atmosphere that prohibits the sale of counterfeit goods. IT experts can identify online sellers, disable the S-commerce platform instantly, and report them to the appropriate authorities. Online platforms are easier to track down unethical trading than offline. Finally, the COVID-19 epidemic has also influenced consumer spending. During the epidemic, both social media use and social commerce business grew. As a result, officials projected greater online buying for counterfeit products. To confront this new condition, the official must formulate policies.

## Limitations and Future Directions

Although this study has some interesting findings and implications, our research has some limitations. First, this study focuses only on attitudes toward the use of counterfeit products rather than intentions and actual behavior. So, future research can be conducted to check the intention and the actual behavior of the consumers. Second, future research could use these dimensions of religiosity (basic duties, central duties, knowledge, experience, and orthodox) on specific counterfeiting products like cosmetics, digital piracy, and online books. Third, many other factors or variables like culture, income, and education should be added to conduct further research. Fourth, this study only focuses on young Muslim consumers. Further studies can consider other religions like Christians, Hindus, Jews, and Buddhism.

## Data Availability Statement

The raw data supporting the conclusions of this article will be made available by the authors, without undue reservation.

## Ethics Statement

Ethical review and approval was not required for the study on human participants in accordance with the local legislation and institutional requirements. Written informed consent from the patients/participants or patients/participants legal guardian/next of kin was not required to participate in this study in accordance with the national legislation and the institutional requirements.

## Author Contributions

SA and HZ: conceptualization and writing of original manuscript. MA and NK: review and improve. PP: data collection and analysis. All authors contributed to the article and approved the submitted version.

## Conflict of Interest

The authors declare that the research was conducted in the absence of any commercial or financial relationships that could be construed as a potential conflict of interest.

## Publisher’s Note

All claims expressed in this article are solely those of the authors and do not necessarily represent those of their affiliated organizations, or those of the publisher, the editors and the reviewers. Any product that may be evaluated in this article, or claim that may be made by its manufacturer, is not guaranteed or endorsed by the publisher.
